# Blinded withdrawal of randomized treatment with low-dose digoxin or placebo in heart failure: the DECISION trial

**DOI:** 10.1093/eurheartj/ehag385

**Published:** 2026-05-10

**Authors:** Peter van der Meer, Dirk J van Veldhuisen, Geert H D Voordes, Jan Tijssen, Marco Alings, Marcel L Bouvy, Henk P Swart, H Jaco Houtgraaf, H G Reinhart Dorman, Michiel Rienstra, Kevin Damman, Arend Mosterd

**Affiliations:** Department of Cardiology, University Medical Centre Groningen, University of Groningen, PO Box 30.001, Groningen 9700RB, The Netherlands; Department of Cardiology, University Medical Centre Groningen, University of Groningen, PO Box 30.001, Groningen 9700RB, The Netherlands; Department of Cardiology, University Medical Centre Groningen, University of Groningen, PO Box 30.001, Groningen 9700RB, The Netherlands; Amsterdam University Medical Center, Amsterdam, The Netherlands; Department of Cardiology, Amphia Hospital, Breda, The Netherlands; Dutch Cardiovascular Research Network (WCN), Utrecht, The Netherlands; Division of Pharmacoepidemiology and Clinical Pharmacology, Utrecht Institute for Pharmaceutical Sciences, Utrecht University, Utrecht, The Netherlands; Department of Cardiology, Antonius Hospital, Sneek, The Netherlands; Dutch Cardiovascular Research Network (WCN), Utrecht, The Netherlands; Department of Cardiology, Diakonessen Hospital, Utrecht, The Netherlands; Dutch Cardiovascular Research Network (WCN), Utrecht, The Netherlands; Department of Cardiology, Bravis Hospital, Roosendaal, The Netherlands; Department of Cardiology, University Medical Centre Groningen, University of Groningen, PO Box 30.001, Groningen 9700RB, The Netherlands; Department of Cardiology, University Medical Centre Groningen, University of Groningen, PO Box 30.001, Groningen 9700RB, The Netherlands; Dutch Cardiovascular Research Network (WCN), Utrecht, The Netherlands; Department of Cardiology, Meander Medisch Centrum, Amersfoort, The Netherlands

## Abstract

**Background and Aims:**

Whether digoxin withdrawal is safe in patients with heart failure (HF) optimized on contemporary guideline-recommended medical therapy remains unknown. This pre-specified analysis of the DECISION trial evaluated outcomes following blinded withdrawal of digoxin or placebo.

**Methods:**

In DECISION, 1001 patients were randomized to low-dose digoxin or placebo and treated for a median of 36.5 months. At the end of the study, 587 patients on active treatment (digoxin 288 and placebo 299) underwent blinded withdrawal with an in-person follow-up visit at 6 weeks. All events were adjudicated.

**Results:**

During the pre-withdrawal phase (100 days), incidence rates of cardiovascular (CV) death or worsening HF events were 5.7 vs 6.5 events per 100 patient-years in the digoxin vs placebo group; rate ratio (RR) 0.88; 95% confidence interval (CI) [0.24–3.10]. Following withdrawal, the incidence rate increased markedly in patients withdrawn from digoxin but not placebo (42.8 vs 5.9 events per 100 patient-years; time period-by-treatment interaction, *P* = .036). Fourteen events (12 hospitalizations and 2 urgent HF visits) occurred in the digoxin withdrawal arm vs two (one HF hospitalization and one CV death) in the placebo withdrawal arm (RR 7.37, 95% CI 1.56–34.88; *P* = .012). Withdrawal of digoxin was accompanied by an increase in heart rate (*P* = .003), reduction in systolic blood pressure (*P* = .014), and rise in NT-proBNP (*P* = .002).

**Conclusions:**

Discontinuation of digoxin after long-term treatment is associated with clinical deterioration in patients with HF and a reduced or mildly reduced ejection fraction. These findings warrant caution when stopping digoxin.

## Structured Graphical Abstract

**Structured Graphical Abstract:**
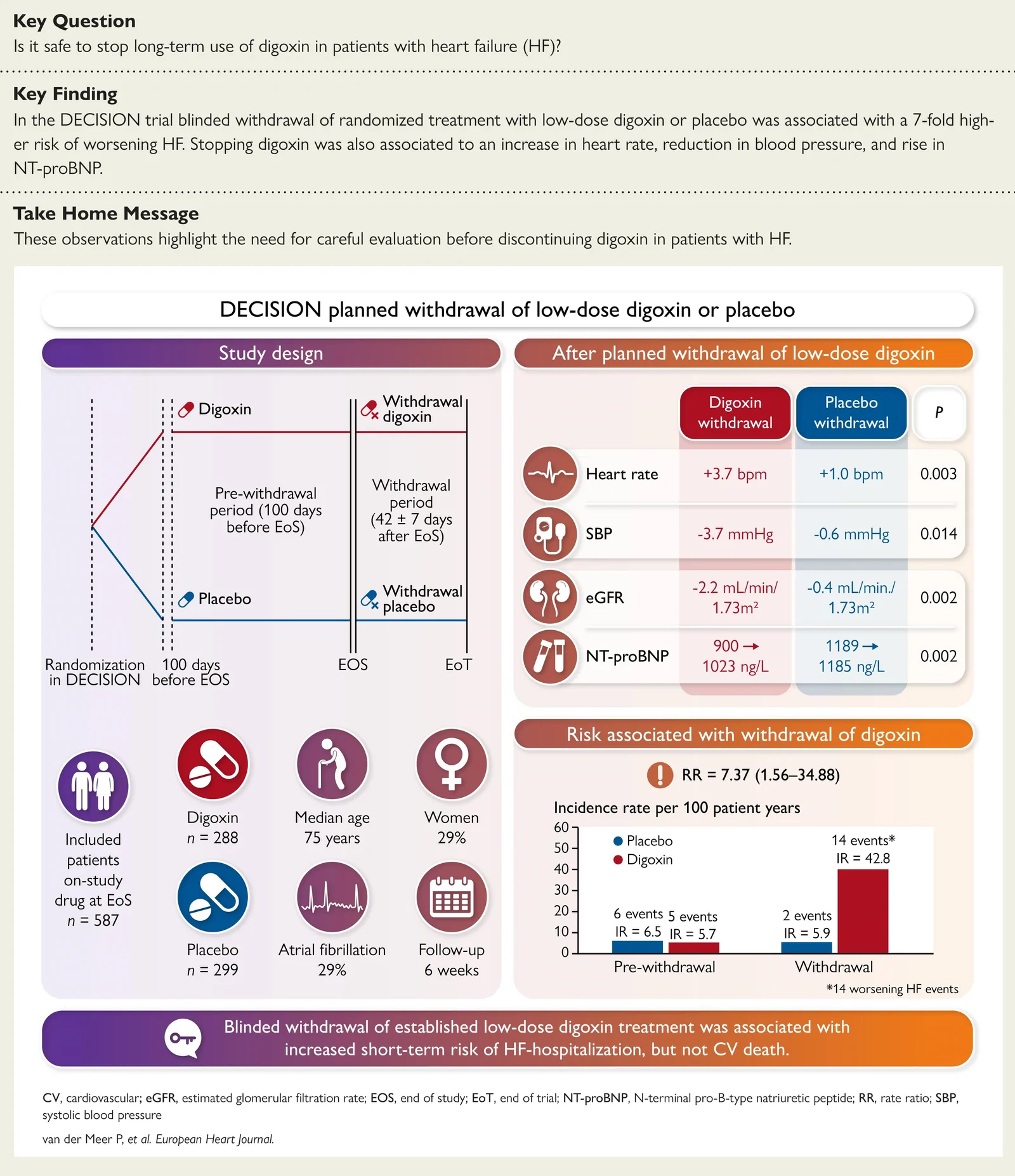
Created in BioRender. Voordes, G.H.D. (2026) https://BioRender.com/uqc4skv

Keywords: Heart failure, Digoxin, Withdrawal

**See the editorial comment for this article ‘Staging a comeback for digitalis’, by L.W. Stevenson and K.T. Murray, https://doi.org/10.1093/eurheartj/ehag629.**

## Introduction

Digitalis glycosides are still playing a significant role in heart failure (HF) management^1,2^ with digoxin currently prescribed to approximately 15%–20% of patients with HF.^3^ Emerging evidence supports the therapeutic value of low-dose cardiac glycosides.^4^ Two recent trials have been executed to study the effect of low-dose cardiac glycosides in the contemporary management of HF. Digitoxin to improve outcome in patients with advanced chronic heart failure (DIGIT-HF) showed that low-dose digitoxin reduced the primary endpoint of heart failure hospitalization and all-cause mortality.^2^ In the Digoxin Evaluation in Chronic Heart Failure: Investigational Study In Outpatients in the Netherlands (DECISION) trial, which included a population of less advanced HF, low-dose digoxin did not result in a significant reduction in the risk of the composite end point of cardiovascular death or recurrent worsening HF events [rate ratio (RR): 0.81; 95% confidence interval (CI) (0.61–1.07), *P* = .133].^5^ A meta-analysis showed that treatment with digitalis glycosides was overall associated with a lower risk of the composite of cardiovascular death or first worsening HF events in patients with HF and a reduced (HFrEF) or mildly reduced ejection fraction (HFmrEF).^6^

However, digoxin is frequently discontinued in clinical practice, often due to concerns about toxicity or perceived redundancy alongside contemporary HF therapies. Whether such withdrawal is safe remains uncertain. Historical studies, from over three decades ago, showed that discontinuing digoxin led to clinical deterioration in patients with HF as recently reported.^7^ However, the relevance of these findings in current clinical practice is unclear, as they were conducted in an era predating modern HF therapies, including beta-blockers, angiotensin receptor-neprilysin inhibitors (ARNIs), sodium-glucose cotransporter-2 inhibitors (SGLT2i), mineralocorticoid receptor antagonists (MRAs), and device therapy. Furthermore, these early studies utilized substantially higher digoxin doses, with serum digoxin concentrations ranging from 0.9 to 2.0 ng/ml, rather than the lower doses used in the most recent trials.^8^

Given the evolving treatment landscape and dosing strategies, the safety of withdrawing long-term, low-dose digoxin therapy remains unclear. We present here a prespecified analysis from the DECISION trial, which examined the effects of blinded withdrawal from low-dose digoxin vs withdrawal of placebo over a 6-week follow-up period.

## Methods

### Study design and patient population

This was a pre-specified analysis in the DECISION study as mentioned in the study protocol. For the planned withdrawal study, we examined all patients that were included in the DECISION-trial and were on-drug at the end of study visit. The design, baseline characteristics, and outcomes of the main DECISION trial have been published before.^5,9,10^ In short, the DECISION trial was an investigator initiated, multicentre, randomized, double-blind placebo-controlled trial that compared low-dose digoxin (0.5–0.9 ng/ml) to placebo in patients with HF with reduced or mid-range ejection fraction (EF ≤ 50%) in the Netherlands. The primary endpoint of DECISION was a combination of repeated HF hospitalizations, repeated urgent HF visits, and cardiovascular death. All participants provided written informed consent and the trial adhered to the declaration of Helsinki.

The end-of-study (EoS) date was set by the steering committee at 1 September 2025, after which an EoS visit was planned for each participant. Participants were instructed to take the last study dose on the day before the EoS visit; this is the start of the withdrawal phase. Patient characteristics and blood samples were collected at this EoS visit. An in-person end-of-trial (EoT) visit was planned 6 weeks (±7 days) after the EoS visit. The period between EoS and EoT is defined as the withdrawal phase. All assessments during the in-person visits were all pre-specified in the DECISION trial protocol (see Supplementary file). However, no formal statistical analysis plan was written for this withdrawal study. Clinical events during the 6-week follow-up period were documented and adjudicated by the blinded independent clinical endpoint committee. Neither patients nor site investigators were informed of treatment allocation prior to, or during the withdrawal follow-up period.

### Outcome

The main events of interest of this study were the combination of cardiovascular mortality, repeated HF hospitalizations and repeated urgent HF visits, which is similar to the primary efficacy endpoint of the trial. In addition, we compared the 100 days before EoS with the 42 days after the EoS. Furthermore, at both visits (EoS and EoT), heart rate and blood pressure were measured together with the following biomarkers: NT-proBNP, creatinine, and eGFR.

### Statistical analysis

Baseline characteristics are presented as mean with standard deviation for normal continuous variables, as median with interquartile ranges (IQR) for non-normal continuous variables or counts with percentages for categorical variables. Variables were compared using Student’s *T*-test for normal distributed variables, Kruskal–Wallis test for non-normal distributed variables and chi-squared test for categorical variables using the Comparegroups-package (version 4.0).

All analyses involving repeated events were analysed using the LWYY semi-parametric proportional rates models for recurrent-event outcomes and the Cox-proportional hazards model for time-to-first-event outcomes.^11^ Estimates for all repeated event analyses are presented as RRs. Estimates for all time-to-first-event outcomes are presented as hazard ratios.

Incidence rates per 100 patient-years were calculated for each treatment group and time period as the total number of events divided by total person-time at risk, with 95% CIs derived using the exact Poisson method. To compare event rates within the digoxin group between the on-treatment and off-treatment periods, a Poisson regression model was fitted with event count as the outcome and the natural logarithm of person-time as an offset term. We calculated an interaction *P*-value to estimate the difference between treatment allocation and time. Estimated marginal means were used to derive the within-digoxin pre- vs post-EoS RR with its 95% CI.

Differences in biomarkers at EoS and EoT were estimated using linear regression models stratified for randomized treatment. Skewed variables were log-transformed using the natural logarithm. Geometric means were obtained by exponentiating the mean of the log-transformed values, with corresponding 95% CIs derived on the log scale and back-transformed. Between-group differences and differences in change over time were assessed on the log scale and expressed as geometric mean ratios with 95% CIs. A *P*-value for interaction was calculated using a linear mixed model with treatment and visit as interaction term and participant as a random effect. All statistical analyses were performed using R/(version 4.2.2) and STATA/SE 19.

## Results

Of the 1001 patients who were initially randomized in DECISION, 288 patients on digoxin and 299 on placebo were taking the study drug at EoS, with a median follow-up of 36.5 months. The CONSORT diagram is shown in Supplementary data online, Figure  *S1*. Baseline pre-randomization characteristics of the patients who entered the withdrawal study (*n* = 587) compared with patients who did not contribute to the withdrawal study (*n* = 414) are shown in Supplementary data online, *Table S1*. Five patients contributed to the pre-withdrawal event rates, but did not participate in the EoS (see Supplementary data online, Figure  *S1*). Patients who participated in the withdrawal study were younger, more often had lower NYHA-class, more frequent valvular disease, more often used ACE, ARNI, and SGLT-inhibitors and had higher eGFR and lower NT-proBNP compared with patients who did not contribute to the withdrawal study.

Patient characteristics at the start of the withdrawal period were overall relatively well balanced between patients on low-dose digoxin vs placebo, with a mean age of 75 years, 29% women and 31.5% of the patients had AF (*Table 1*). Median digoxin concentration at the start of withdrawal period was 0.6 ng/ml (IQR 0.5–0.7), with two of the patients (0.7%) having a SDC >1.2 ng/ml. The overall use of guideline recommended medical therapy was high. In total, 86% were on beta-blockers, 88% on an ACE inhibitor/ARB/ARNI, 73% on an MRA, and 72% on an SGLT2 inhibitor. Guideline-recommended medical therapy was not different between low-dose digoxin withdrawal or placebo withdrawal, except for the use of SGLT2i, which was more prevalent in patients on placebo.

**Table 1:** Summary statistics of the withdrawal of digoxin study at the time of end of study

	All	Digoxin	Placebo	*P*.overall
	*N* = 587	*N* = 288	*N* = 299	
Age at EoS (yrs ± SD)	75.2 (8.68)	75.3 (8.86)	75.0 (8.51)	.702
Sex (% female)	172 (29.3%)	82 (28.5%)	90 (30.1%)	.732
LVEF at randomization (%± SD)	32.7 (9.20)	33.4 (9.40)	32.0 (8.97)	.058
	149 (25.4%)	73 (25.4%)	76 (25.4%)	1.000
	27.8 (4.97)	28.1 (4.92)	27.6 (5.02)	.262
NYHA-class at EoS (%)				.769
II	535 (91.1%)	264 (91.7%)	271 (90.6%)	
III	52 (8.86%)	24 (8.33%)	28 (9.36%)	
SBP at EoS (mmHg ± SD)	124 (18.7)	126 (18.3)	121 (18.7)	.001
DBP at EoS (mmHg ± SD)	71.0 (10.3)	70.7 (10.1)	71.3 (10.5)	.489
BMI at EoS (kg/m^2^ ± SD)	26.8 (4.74)	26.5 (4.64)	27.2 (4.81)	.074
Heart rate at EoS (bpm ± SD)	68.8 (11.3)	67.0 (11.3)	70.5 (11.1)	<.001
Atrial fibrillation at EoS (%)	181 (31.5%)	90 (31.9%)	91 (31.1%)	.895
Ventricular rhythm at EoS				.891
Biventricular pacing	102 (17.7%)	48 (17.0%)	54 (18.4%)	
Intrinsic conduction	413 (71.8%)	205 (72.7%)	208 (71.0%)	
Right Ventricular pacing	60 (10.4%)	29 (10.3%)	31 (10.6%)	
Digoxin concentration at EoS (ng/ml ± SD)	0.63 (0.21)	0.63 (0.21)	NA	
Betablocker at EoS (%)	508 (86.5%)	248 (86.1%)	260 (87.0%)	.858
ACE, ARB or ARNI at EoS (%)	515 (87.7%)	258 (89.6%)	257 (86.0%)	.225
MRA at EoS (%)	428 (72.9%)	204 (70.8%)	224 (74.9%)	.308
SGLT2-inhibitor at EoS (%)	425 (72.4%)	195 (67.7%)	230 (76.9%)	.016
Creatinin at EoS (µmol/L ± IQR)	108 [88.0;136]	106 [89.0;132]	111 [88.0;138]	.219
eGFR at EoS (ml/min/1.73 m^2^ ± IQR)	54.0 [41.8;67.0]	56.0 [43.0;68.0]	52.0 [41.0;66.5]	.196
NTproBNP at EoS (ng/L ± IQR)	1121 [518;2258]	1013 [397;2176]	1270 [660;2271]	.004

ACE, angiotensin-converting enzyme; ARB, angiotensin receptor blocker; ARNI, angiotensin receptor and neprilysin inhibitor; BMI, body mass index; DBP, diastolic blood pressure; eGFR, estimated glomerular filtration rate; EoS, end of study; IQR, interquartile range; kg, kilograms; LVEF, left ventricular ejection fraction; MRA, mineralocorticoid receptor antagonist; NYHA, New York Heart Association; SBP, systolic blood pressure; SD, standard deviation; SGLT2-I, sodium glucose co-transporter 2 inhibitor.

At the start of the withdrawal period, patients on low-dose digoxin had a significantly higher systolic blood pressure (126 ± 18 mmHg vs 121 ± 19 mmHg; *P* = .001), a significantly lower heart rate (67 ± 11 bpm) vs (71 ± 11 bpm), *P* < .001, and lower NT-pro BNP levels [1013 (397–2176) vs 1270 (660–2271) ng/L, *P* = .004] with a similar rate of patients with atrial fibrillation (32% vs 31%). Kidney function was similar between both groups.

The median follow time between start of withdrawal and end of withdrawal study was 43 days (IQR 41–44). No patients were lost to follow-up. During this period, 14 events of interest (CV death or recurrent HF hospitalizations or urgent HF visits) occurred in patients withdrawn from low-dose digoxin, compared with two events in patients withdrawn from placebo (RR 7.37, 95% CI 1.56–34.88; *P* = .012) (*Figure 1A*, *Table 2*). The composite signal was driven entirely by worsening HF; recurrent HF hospitalizations and urgent HF visits occurred 14 times in the digoxin withdrawal group, of which 12 were HF hospitalizations and two were urgent HF visits, compared with one HF hospitalization in the placebo withdrawal group RR 14.77, 95% CI 1.85–118.26, *P* = .011) (*Figure 1B* and *Table 2*). One patient in the placebo withdrawal group died from a cardiovascular cause, where no cardiovascular deaths occurred in the low-dose digoxin withdrawal group. Finally, there was a single non-CV death in both groups. For the time-to-first event, 10 unique patients in the digoxin withdrawal arm experienced an event vs two in the placebo withdrawal arm. The absolute risk difference is 2.8%.

**Figure 1:**
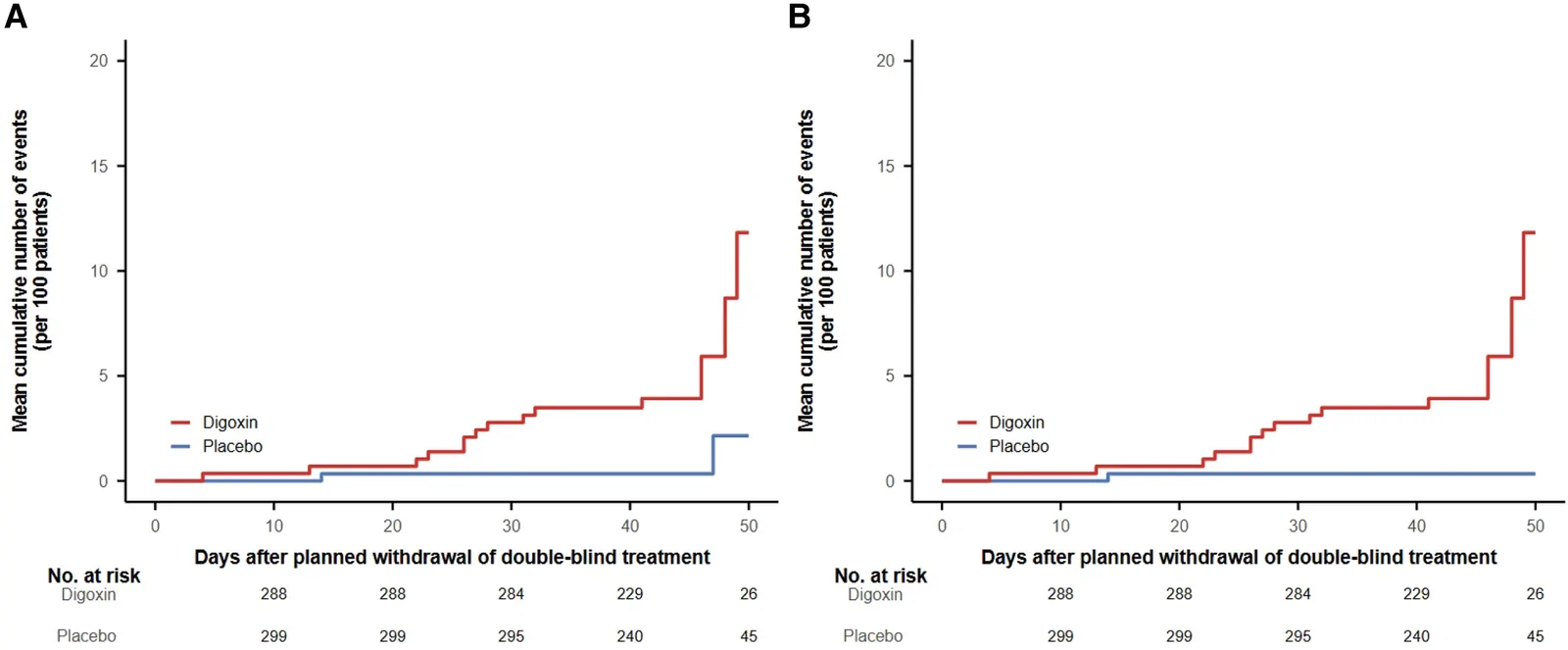
Panel figure showing the cumulative incidence of repeated events in either the digoxin group or the placebo group. *Figure 1A* shows the combined endpoint of cardiovascular death, heart failure hospitalizations and urgent heart failure (HF) visits, while *Figure 1B* shows the combined endpoint of heart failure hospitalizations and urgent HF visits

**Table 2:** Clinical events during withdrawal period

Analysis	Events Digoxin (n)	Incidence rate/100 patient years Digoxin	Events Placebo (n)	Incidence rate/100 patient years Placebo	RR/HR	*P*-value
Combination of cardiovascular death, recurrent HF hospitalizations and urgent HF visits	14	42.81	2	5.85	7.37 [1.56–34.88]	.012
Combination of recurrent HF hospitalizations and urgent HF visits	14	42.81	1	2.93	14.77 [1.85–118.26]	.011
Cardiovascular death	0	0	1	2.86	NA	NA
Combination of recurrent cardiovascular hospitalizations and cardiovascular visits	2	6.29	5	14.8	0.42 [0.08–2.36]	.327
Time-to-first-event heart failure hospitalization, urgent HF visits, or cardiovascular death	10	30.31	2	5.73	5.52 [1.21–25.22]	.028
Time-to-first-event heart failure hospitalization, urgent heart failure visits, or all-cause death	11	33.34	3	8.59	4.03 [1.12–14.45]	.033
Time-to-first heart failure hospitalization, urgent heart failure visits	10	30.31	1	2.86	10.83 [1.39–84.69]	.023
Time-to-all cause death	1	3.03	2	5.73	0.58 [0.05–6.37]	.653

During the 100 days in the pre-withdrawal phase, the incidence rate of cardiovascular death or worsening heart failure events was 5.7 events per 100 patients-years in the low-dose digoxin group and 6.5 events per 100 patient-years in the placebo group; RR = 0.88, 95% CI (0.24–3.10) (*Figure 2*). In the withdrawal phase, the incidence rate of cardiovascular death or worsening heart failure increased in patients withdrawn from low-dose digoxin but not in those withdrawn from placebo (42.8 events per 100 patient-years and 5.9 events per 100 patient-years, respectively). The RR for the change in risk in the patients withdrawn from low-dose digoxin was 7.55 (95% CI, 2.72–20.95), *P* < .001, whereas the change in the risk in patients withdrawn from placebo was not significant [RR = 0.89 (95% CI, 0.18–4.44)]; time period-by-treatment interaction, *P* = .036.

**Figure 2:**
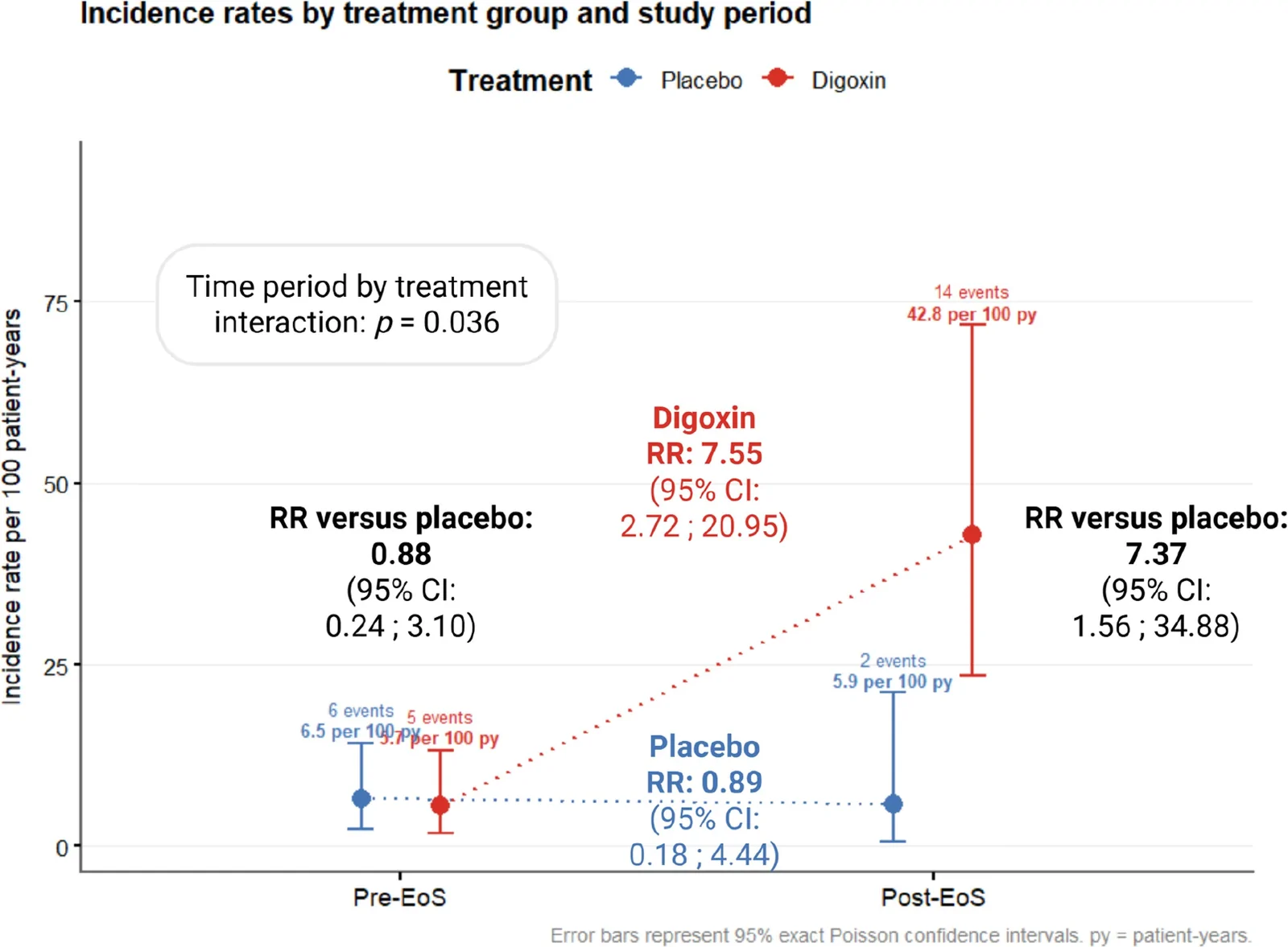
Cardiovascular risk before and during withdrawal of low-dose digoxin and placebo. Incidence rate of cardiovascular death and worsening heart failure per 100 patient-years

*Table 3* shows the changes in clinical variables and biomarkers during the withdrawal period, including the existing baseline differences. Withdrawal of low-dose digoxin was associated with in a higher heart rate (*P* = .003) and lower systolic blood pressure (*P* = .014) (*P* for interaction = .003 and .017, respectively). Withdrawal of low-dose digoxin was associated with an increase in NT-proBNP levels compared with withdrawal from placebo (*P* = .002, *P*-value for interaction = .002). Furthermore, eGFR declined significantly more in patients withdrawn from low-dose digoxin compared with withdrawal from placebo (*P* = .001, *P*-value for interaction .002). These effects were present independently of the underlying heart rhythm, except for changes in NT-proBNP levels, which were only observed in patients in sinus rhythm (see Supplementary data online, *Table S3*).

**Table 3:** Clinical and biomarker changes during the withdrawal period

	Visit	Withdrawal of Digoxin	*N*	Withdrawal of Placebo	*N*	GMR or difference	*P-*value	*P-*int
**NTproBNP (ng/L)**	EoS	900 (779–1041)^a^		1189 (1051–1344)^a^		0.76 (0.63–0.92)^b^	.004	.002
	EoT	1023 (889–1177)^a^		1185 (1040–1352)^a^		0.86 (0.71–1.04)^b^	.132	
	Geometric mean ratio (EoT-EoS)	1.14 (1.06–1.21)^b^	259	1.00 (0.95–1.05)^b^	260	1.14 (1.05–1.24)^b^	.002	
**eGFR (ml/min/1.7 3 m^2^)**	EoS	56.2 (17.2)		53.7 (17.6)		2.5 (25)	.099	.001
	EoT	54.0 (16.7)		53.4 (17.5)		0.7 (24)	.656	
	Difference (EoT-EoS)	−2.2 (7.1)	269	−0.4 (6.0)	280	−1.8 (16)	.001	
**Heart rate (bpm)**	EoS	67.0 (11.3)		70.5 (11.1)		−3.5 (16)	<.001	.003
	EoT	70.6 (12.0)		71.5 (11.9)		−0.9 (17)	.373	
	Difference (EoT-EoS)	3.7 (10.9)	277	1.0 (10.2)	281	2.6 (15)	.003	
**SBP (mmHg)**	EoS	126.5 (18.4)		121.3 (18.7)		5.2 (26)	.001	.014
	EoT	122.7 (18.1)		120.7 (18.4)		2.0 (26)	.193	
	Difference (EoT-EoS)	−3.7 (15.2)	274	−0.6 (14.88)	282	−3.1 (21)	.014	

Shown are mean (standard deviation) unless indicated otherwise. Analyses were conducted in patients with data available for each variable at both endpoints. Therefore, the number of cases available for analysis at EoS, EoT, and the difference between both are the same within each treatment arm.

^a^Geometric mean (95% confidence interval).

^b^Geometric mean ratio (95% confidence interval).

## Discussion

In this pre-specified blinded withdrawal analysis from the DECISION trial, discontinuation of long-term low-dose digoxin was associated with a clinically meaningful and statistically significant increase in the composite of cardiovascular death, recurrent HF hospitalization, and urgent HF visits compared with withdrawal from placebo. Over a relatively short 6-week follow-up period, patients withdrawn from digoxin experienced a more than seven-fold higher rate of these events driven by HF-related hospitalizations. These findings were associated with a parallel deterioration in physiological and biochemical markers.

The results of the withdrawal study have to be seen in the context of the DECISION main trial; which demonstrated a non-significant (i.e. neutral) effect of low-dose digoxin on the primary composite endpoint of cardiovascular death, recurrent HF hospitalization, and urgent HF visits [RR: 0.81 95% CI (0.61–1.07), *P* = .133].^5^ Although the event rate in DECISION was as assumed, an important aspect of the main results of the DECISION study was the relatively high drop-out rate, which has led to a loss of statistical power. At the end of the study, 92 of 382 patients (24%) in the digoxin group and 77 of 375 patients (21%) in the placebo group had discontinued study drug treatment for reasons other than death. Interestingly, this did not seem to be related to digoxin-associated adverse events. To correct for this relatively high discontinuation rate, an as-treated sensitivity analysis was also performed. In this analysis, an RR of 0.66 (95% CI 0.47–0.92, *P* = .015) was observed. This as-treated analysis is more in line with the results of the withdrawal study, where only patients on study drug have been investigated.

An important aspect of the withdrawal study is that clinical deterioration was observed despite the high use of contemporary HF therapies. At the time of withdrawal, 86% of patients were on beta-blockers, 88% on an ACE inhibitor/ARB/ARNI, 73% on an MRA, and 72% on an SGLT2 inhibitor. It has been suggested that the haemodynamic and neurohormonal benefits of these newer HF drugs would limit the effect of digoxin.^12^ Our data suggest this is not the case. The contribution of low-dose digoxin to haemodynamic stability, heart rate control, and neurohormonal modulation appears to persist even in the context of optimized background therapy.

The finding that withdrawal of HF therapy triggers clinical deterioration is not unique to digoxin. In the EMPEROR trials, blinded withdrawal of empagliflozin was associated with increased risk of major HF events,^13^ with physiological changes that were the inverse of those seen during treatment initiation, and the FINEARTS-HF withdrawal analysis similarly showed that discontinuation of finerenone resulted in increased cardiovascular risk.^14^ In TRED-HF, even among patients with fully recovered ejection fraction, 44% relapsed after withdrawal of all pharmacological HF treatment.^15^ Across distinct drug classes, these data consistently support caution before elective discontinuation of established HF therapy in stable patients.

The consequences associated with digoxin discontinuation are consistent with findings from earlier withdrawal trials; however, important differences exist. The 1993 RADIANCE trial randomized 178 stable HF patients on diuretics, ACE inhibition, and digoxin (LVEF <35%) to continuation of digoxin or placebo, and reported a relative risk of 5.9 (95% CI 2.1–17.2) for worsening heart failure.^16^ The 1993 PROVED trial similarly showed a significant increase in worsening heart failure events during digoxin withdrawal in 88 stable patients on diuretics during 12 weeks follow-up (39%, digoxin withdrawal group vs 19%, digoxin maintenance group).^17^ The magnitude of excess risk observed in our study is similar to the RADIANCE study, despite a fundamentally different background treatment and serum digoxin concentrations. In these trials SDC were substantially higher (0.9–2.0 ng/ml) than the low-dose target in DECISION (median SDC 0.6 ng/ml), indicating that even a low-dose digoxin has an important clinical effect. Further evidence comes from a *post hoc* analysis of 3365 patients enrolled in the 1997 DIG trial who were taking digoxin at baseline. Among those randomized to placebo and, as such effectively withdrawn from digoxin, rates of HF and total hospitalization and all-cause mortality were higher compared with those receiving low-dose digoxin, the same target concentration range used in DECISION.^8^

RADIANCE and PROVED enrolled only patients in sinus rhythm, leaving uncertainty about whether withdrawal effects generalize to patients with AF. A small, randomized withdrawal trial (*n* = 47) of HF patients with AF (mean LVEF 24%) receiving ACE inhibition, carvedilol, and digoxin demonstrated an increase in BNP levels and a decline in LVEF when stopping digoxin, though without a significant increase in clinical symptoms.^18^ In the present DECISION withdrawal cohort, approximately one-third of patients had AF at baseline. The changes in clinical variables and biomarkers were observed across the group as a whole and no significant interaction was observed between sinus rhythm and AF except for NT-proBNP levels, which increased only in patients with sinus rhythm after withdrawal of low-dose digoxin. However, the event-rate is too low to draw rhythm-specific conclusion.

The unfavourable clinical and biomarker changes observed following digoxin withdrawal may provide some mechanistic insights. The observed increase in NT-proBNP, together with the rise in heart rate and decline in blood pressure and eGFR, may be related to the discontinuation of the beneficial neurohormonal and haemodynamic effects of digoxin.^19,20^ The increase in heart rate, both in patients with sinus rhythm and AF, observed after digoxin withdrawal, could be related to the effects on vagal tone.^21^

There are several limitations. First, the withdrawal study population (*n* = 587) represents only those patients who survived and remained on study drug at the end of the main DECISION trial, approximately 59% of those originally randomized. As shown in Supplementary data online, Table  *S1*, these patients were younger, more frequently NYHA II, had lower NT-proBNP, better renal function and higher use of SGLT2 inhibitors than those who did not enter the withdrawal phase. This survival and adherence bias means the withdrawal cohort is inherently a selected, lower-risk population who tolerated and remained stable on long-term low-dose digoxin. The effects of digoxin withdrawal in sicker or less well-treated patients remains unknown. Furthermore, baseline differences at the start of the withdrawal phase are present between the patients on low-dose digoxin compared with placebo. Second, although this was a pre-specified analysis embedded within a randomized controlled trial, the withdrawal phase itself was not powered as a standalone endpoint study. Third, the DECISION trial was conducted exclusively in the Netherlands, and the cohort was predominantly Caucasian. Generalization of these findings to non-European populations should therefore be made with caution. Fourth, no data on quality of life were collected during the withdrawal phase and such data would have strengthened the interpretation of whether the biomarker changes translated into meaningful changes from the patient’s perspective. Finally, our findings relate to patients maintained on long-term low-dose digoxin and do not address the question of *de novo* initiation. Furthermore, while our data argue against routine withdrawal, they do not define specific clinical circumstances, for example, side effects or severely impaired renal function, in which stopping digoxin may still be appropriate.

## Conclusion

Discontinuation of long-term low-dose digoxin is associated with clinical deterioration of patients with chronic heart failure and a left ventricular ejection fraction 50%. These findings warrant caution when stopping low-dose digoxin ≤ 50%.

## Supplementary Material

ehag385_Supplementary_Data

## Declarations

### Data Availability

The datasets generated and/or analysed during this study are not publicly available, but anonymized participant data can be made available upon requests directed to the corresponding author. The steering committee of DECISION will review requests and will have the right to review and comment on any draft papers based on these data before publication. Data requests will be reviewed on the basis of scientific merit, overlap with any planned secondary analysis, ethical review, available resources and regulatory requirements, and a response can be expected within 2 weeks. Following approvals, unidentified patient data and a data dictionary will be provided.

### Pre-registered Clinical Trial Number

NCT03783429.
