# Supplementary material for: Blinded withdrawal of randomized treatment with low-dose digoxin or placebo in heart failure: the DECISION trial
Source: Eur Heart J. 2026 May 10;47(38):5545–53. doi: 10.1093/eurheartj/ehag385 (PMC13651696; doi:10.1093/eurheartj/ehag385)

Suppl Figure 1 CONSORT diagram

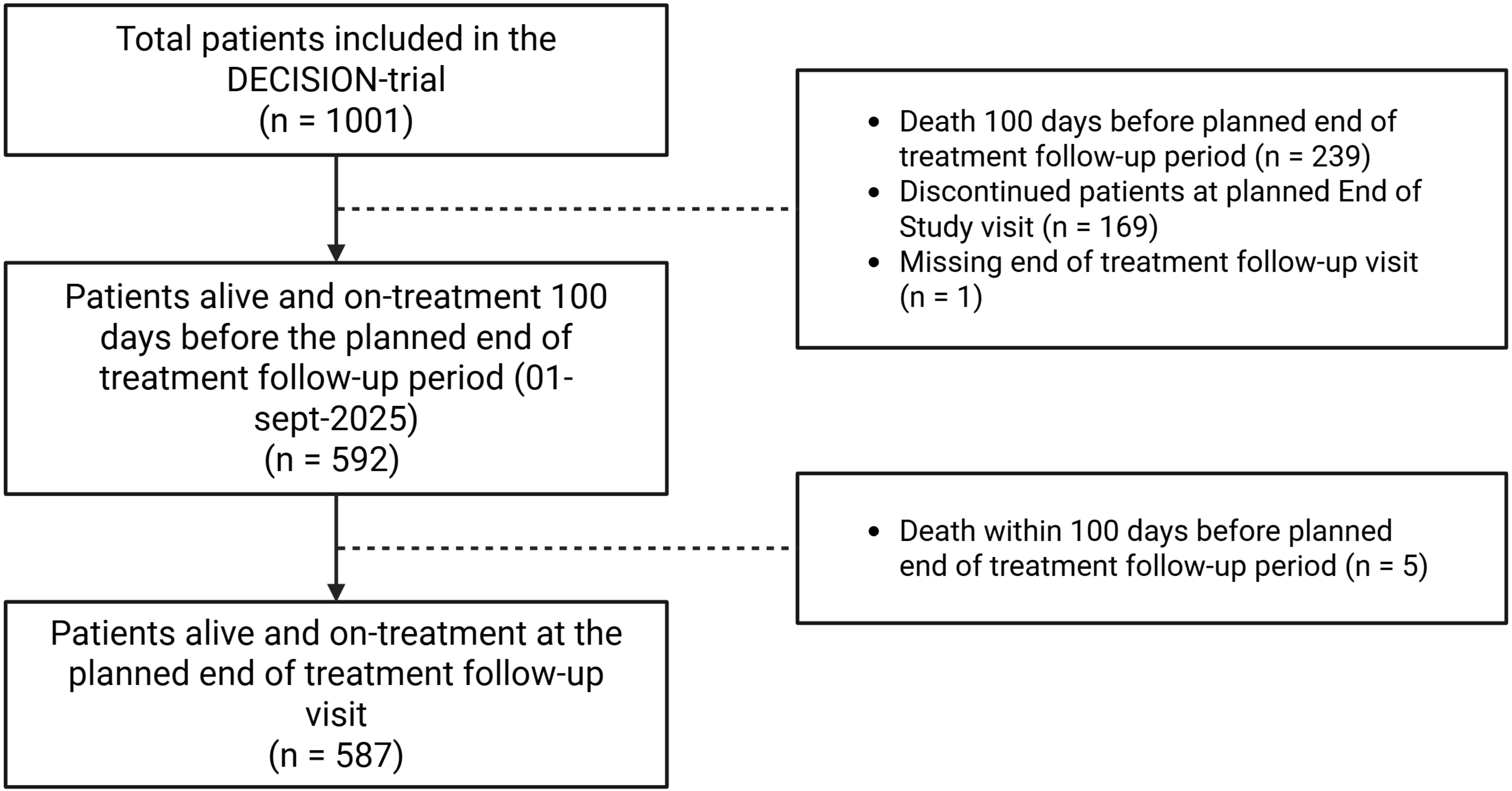

Supplement: Supplementary file 1 [file ehag385_supplementary_data.zip › suppl. figure 1.pdf]
