# Supplementary material for: Blinded withdrawal of randomized treatment with low-dose digoxin or placebo in heart failure: the DECISION trial
Source: Eur Heart J. 2026 May 10;47(38):5545–53. doi: 10.1093/eurheartj/ehag385 (PMC13651696; doi:10.1093/eurheartj/ehag385)
Supplement: Supplementary file 1 [file ehag385_supplementary_data.zip › Supplementary tables.docx]

**Supplementary**

Supplementary table 1: Measurements at baseline of patients included in the withdrawal study and patients not included in the withdrawal study.

|  | **Not in withdrawal study** | **In withdrawal study** | **p.overall** |
| --- | --- | --- | --- |
|  | ***N=414*** | ***N=587*** |  |
| Age (yrs.) | 73.4 (8.45) | 71.8 (8.67) | 0.003 |
| Sex (% female) | 112 (27.1%) | 172 (29.3%) | 0.480 |
| BMI (kg/m2) | 27.3 (4.88) | 27.8 (4.97) | 0.086 |
| LVEF (%) | 33.5 (9.02) | 32.7 (9.20) | 0.188 |
| NYHA-class (%) |  |  | <0.001 |
| II | 341 (82.4%) | 535 (91.1%) |  |
| III | 72 (17.4%) | 52 (8.86%) |  |
| IV | 1 (0.24%) | 0 (0.00%) |  |
| Heart rate (bpm) | 73.6 (12.7) | 72.3 (12.2) | 0.118 |
| Systolic blood pressure (mmHg) | 123 (19.5) | 124 (18.7) | 0.321 |
| History of Valvular disease (%) | 131 (31.6%) | 134 (22.9%) | 0.002 |
| Diabetes Mellitus (%) | 117 (28.3%) | 149 (25.4%) | .354 |
| COPD (%) | 61 (14.7%) | 60 (10.2%) | 0.040 |
| Atrial fibrillation/-flutter (%) | 122 (29.5%) | 172 (29.3%) | 1.000 |
| Hemoglobin (mmol/L) | 8.60 [7.80;9.30] | 8.80 [8.20;9.50] | <0.001 |
| NTproBNP (ng/L) | 1610 [1012;2791] | 1295 [908;2016] | <0.001 |
| eGFR (ml/min/1.73m2) | 55.1 (17.0) | 58.1 (15.6) | 0.004 |
| eGFR_below_60 (%) | 274 (66.2%) | 314 (53.5%) | <0.001 |
| Betablocker (%) | 351 (84.8%) | 516 (87.9%) | 0.182 |
| ACE/ARB/ARNI (%) | 363 (87.7%) | 533 (90.8%) | 0.138 |
| Diuretics (%) | 323 (78.0%) | 418 (71.2%) | 0.019 |
| MRA (%) | 285 (68.8%) | 436 (74.3%) | 0.069 |
| SGLT2i (%) | 141 (34.1%) | 272 (46.3%) | <0.001 |

Supplementary table 2

Stratification of clinical and biomarker variables for sinus rhythm and atrial fibrillation

| **Parameter** |  | **Row** | **N** | **Digoxine**  **(Mean ± SD / IQR)** | **Placebo**  **(Mean ± SD / IQR)** | **Difference**  **(Digoxine-Placebo)** | ***p*-value** | ***p-*interaction** | ***p*-interaction**  **Visit*Med*AF** |
| --- | --- | --- | --- | --- | --- | --- | --- | --- | --- |
| NTproBNP* | SR | EoS | 355 | 649 [545–772] | 991 [853–1150] | 0.65 [0.52–0.82] | <0.001 | 0.002 | <0.001 |
|  |  | EoT |  | 792 [663–945] | 1026 [873–1205] | 0.77 [0.61–0.98] | 0.035 |  |  |
|  |  | Ratio EoT/EoS (within group) |  | 1.22 [1.12–1.32] | 1.04 [0.97–1.10] | 1.18 [1.06–1.31] | 0.002 |  |  |
|  | AF | EoS | 162 | 1812 [1499–2190] | 1746.3 [1437–2121] | 1.04 [0.79–1.36] | 0.791 | 0.442 |  |
|  |  | EoT |  | 1754 [1475–2085] | 1603.9 [1298–1982] | 1.09 [0.83–1.44] | 0.523 |  |  |
|  |  | Ratio EoT/EoS (within group) |  | 0.97 [0.88–1.06] | 0.92 [0.83–1.01] | 1.05 [0.92–1.21] | 0.446 |  |  |
| eGFR | SR | EoS | 375 | 58.0 (17.7) | 54.3 (18.3) | 3.71 (25.5) | 0.047 | 0.018 | 0.442 |
|  |  | EoT |  | 56.0 (17.4) | 53.9 (18.3) | 2.1 (25.2) | 0.266 |  |  |
|  |  | Difference EoT - EoS |  | -2.0 (7.2) | -0.4 (6.2) | -1.6 (9.5) | 0.018 |  |  |
|  | AF | EoS | 172 | 52.5 (15.4) | 52.6 (16.0) | -0.1 (22.2) | 0.950 | 0.017 |  |
|  |  | EoT |  | 49.9 (14.5) | 52.3 (16.0) | -2.4 (21.3) | 0.298 |  |  |
|  |  | Difference EoT - EoS |  | -2.5 (6.8) | -0.3 (5.4) | -2.2 (8.7) | 0.017 |  |  |
| Heart rate | SR | EoS | 381 | 66.0 (11.1) | 68.5 (10.1) | -2.5 (15.1) | 0.023 | 0.095 | 0.134 |
|  |  | EoT |  | 68.9 (11.8) | 69.6 (10.7) | -0.7 (15.9) | 0.535 |  |  |
|  |  | Difference EoT - EoS |  | 2.8 (11.2) | 1.0 (9.6) | 1.8 (14.8) | 0.096 |  |  |
|  | AF | EoS | 177 | 68.9 (11.3) | 74.7 (12.0) | -5.8 (16.5) | 0.001 | 0.006 |  |
|  |  | EoT |  | 74.4 (11.5) | 75.7 (13.4) | -1.3 (17.6) | 0.482 |  |  |
|  |  | Difference EoT - EoS |  | 5.5 (9.8) | 1.0 (11.3) | 4.4 (15.0) | 0.006 |  |  |
| SBP | SR | EoS | 381 | 126.8 (18.7) | 120.4 (18.9) | 6.4 (26.6) | <0.001 | 0.002 | 0.180 |
|  |  | EoT |  | 122.6 (18.4) | 121.0 (18.1) | 1.6 (25.8) | 0.385 |  |  |
|  |  | Difference EoT - EoS |  | -4.1 (15.4) | 0.6 (15.2) | -4.8 (21.6) | 0.002 |  |  |
|  | AF | EoS | 174 | 125.8 (18.0) | 123.5 (18.2) | 2.3 (25.6) | 0.401 | 0.745 |  |
|  |  | EoT |  | 123.1 (17.5) | 120.1 (19.0) | 3.0 (25.8) | 0.279 |  |  |
|  |  | Difference EoT - EoS |  | -2.7 (14.9) | -3.4 (13.6) | 0.7 (20.15) | 0.746 |  |  |

**Suppl Figure 1**: CONSORT diagram
